# Increased autophagy in EOC re-ascites cells can inhibit cell death and promote drug resistance

**DOI:** 10.1038/s41419-018-0449-5

**Published:** 2018-03-16

**Authors:** Yu Liu, Jing Tang, Duanyang Liu, Lei Zhang, Yan He, Jing Li, Lei Gao, Dai Tang, Xiaoming Jin, Dan Kong

**Affiliations:** 1 Department of Pathology, Harbin Medical University, Harbin, China; 2 Electron Microscopic Center, Harbin Medical University, Harbin, China; 3 Department of Gynecology, Tumor Hospital of Harbin Medical University, Harbin, China

## Abstract

As the major and preferred treatment for ovarian cancer ascites, chemotherapy can reduce or inhibit recurrent ascites (hereafter re-ascites); however, some patients still experience re-ascites. Therefore, this study investigated cases in which epithelial ovarian cancer (EOC) patients experienced re-ascites. In re-ascites cases, CA125, MDR1, LC-3, and Beclin-1 were highly expressed. In addition, CASP-9 and c-CASP-3 expression levels were decreased, and serum CA125 levels (highest 4348 U/ml) were increased compared to chemosensitive cases. The results suggest that high expression levels of Beclin-1 and LC-3, thus increasing the level of autophagy and inhibiting apoptosis in the no-chemotherapy group. In the chemosensitive group, survivin expression was decreased and CASP-9 expression was increased, which led to c-CASP-3 activation and increased tumor cell apoptosis. The results of the cell lines confirm that inhibition of autophagy can increase the sensitivity of ovarian cancer cells to CDDP and promote CDDP-induced cell death. Re-ascites, which appears after chemotherapy, may be associated with drug resistance. In addition, increased autophagy may protect tumor cells from chemotherapeutic drugs, thus inhibiting tumor cell death.

## Introduction

Epithelial ovarian cancer (EOC) is one of the most common gynecological malignancies with a 5-year survival rate lower than 30%; EOC is often accompanied by ascites in late stages^1,2^. The preferred clinical treatment of ovarian cancer accompanied with ascites is chemotherapy after surgery. The conventional chemotherapy drug is cisplatin, a platinum agent, combined with paclitaxel to inhibit or kill tumor cells. For chemosensitive patients, conventional chemotherapy can inhibit ascites, improve survival quality, and prolong survival time. However, some patients treated with chemotherapy still experience re-ascites, and the recurrence rate is increasing, which is a significant problem in the treatment of advanced ovarian cancer patients^3,4^.

Ovarian cancer ascites is caused by the abdominal spread of tumor cells. The typical characteristics of malignant tumors are fast proliferation and strong invasion. Thus, blood supply frequently cannot meet the growth of the tumor, resulting in a stressed tumor microenvironment that is low in oxygen and deficient in nutrient. Cancer cells survive in such stressful environments by activating various signals, such as the unfolded protein response, changing the metabolic pathway, and undergoing autophagy^5,6^. The effect of autophagy on tumor energy metabolism provides a theoretical basis for the survival mechanism of tumors under stress conditions.

Autophagy is a type of cellular catabolic degradation response to nutrient starvation or metabolic stress^7^ and is a double-edged sword in tumorigenesis and metastasis. Excessive autophagy can induce autophagic cell death;^8^ in contrast, autophagy can also play a protective role in tumor cells. Cancers can use autophagy-mediated recycling to maintain mitochondrial function and energy homeostasis to meet the elevated metabolic demand of growth and proliferation, thus resulting in drug resistance^9^. Autophagy can also affect the biological behavior of the tumor by influencing glucose uptake, glycolysis, oxidative phosphorylation, lipid metabolism, and amino acid metabolism in the tumor cell^10–12^.

Various autophagy-related proteins play important roles in the process of autophagy. Microtubule-associated protein 1 light chain 3 (LC-3) is an important gene involved in autophagy. Beclin-1 acts as a tumor suppressor gene to regulate and promote autophagy by enhancing PI3KC3 kinase activity, thereby inhibiting tumor growth^13^. However, autophagy can also play a pro-tumor role in carcinogenesis by regulating a number of pathways, including Beclin-1, Bcl-2, Class III and I PI3K, mTORC1/C2, and p53^14,15^. This study investigated the relationships among chemoresistance, autophagy, and apoptosis by evaluating ovarian cancer ascites cases. In addition, we compared the differences between the no-chemotherapy and chemosensitive groups to better understand the processes regulating cell autophagy, which may reveal potential therapeutic targets for drug-resistant tumors.

## Results

### Case analysis of EOC patients with re-ascites

**Fig. 1:**
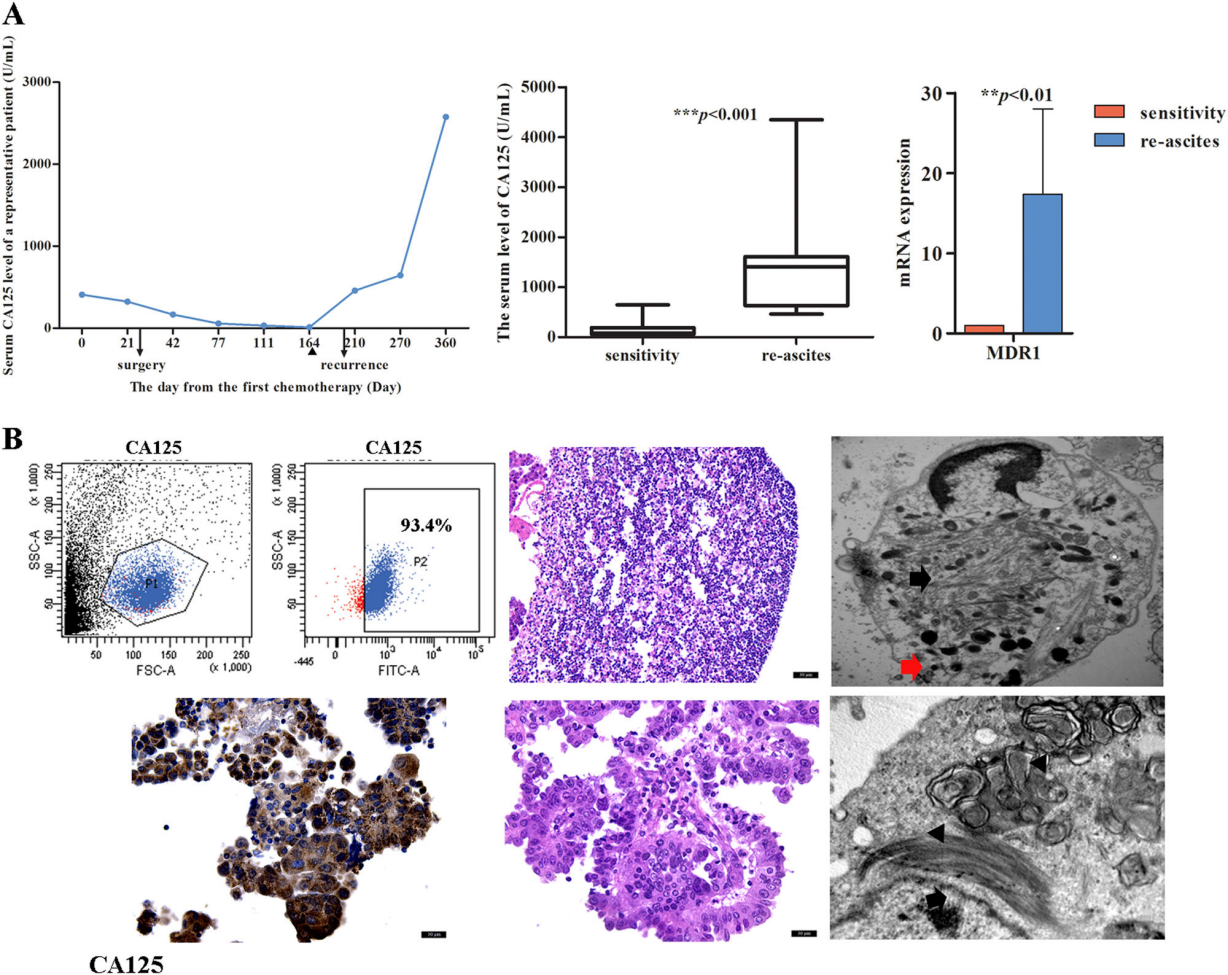
Characteristics of EOC re-ascites. **a** As the time and number of chemotherapy treatments increased, serum CA125 levels also increased, and serum CA125 levels and MDR1 mRNA levels in the re-ascites group were higher than those in the chemosensitive group. The triangle indicates the time point at which the patient finished treatment and left the hospital. (***p < 0.001 and **p < 0.01.) **b** The number of precipitated ascites tumor cells was increased, and many microfilaments (arrow in black), primary lysosomes (arrow in red), and myelin figures (triangle) were visible

Among 45 cases in which ascites developed after chemotherapy, 20 involved chemoresistant patients (Fig. S1). Analysis of clinicopathological data found that serum CA125 levels, which tend to return to a normal range after chemotherapy, were increased during re-ascites (as high as 4348 U/ml), and the difference was statistically significant (Fig. 1a, *p* < 0.001). Meanwhile, 45% (9/20) of tumor tissues were positive for MDR expression. In 25 chemosensitive patients, the serum CA125 level was reduced to a normal level, and ascites were not observed. The qRT-PCR results confirmed that the MDR1 expression level in the re-ascites group was significantly higher than that in the chemosensitive group (Fig. 1a, *p* < 0.01). Detection of re-ascites revealed tumor cell contents as high as 93.4% and high CA125 expression levels, as determined by IHC. Under a light microscope, tumor cells were shown to be densely arranged with a visible glandular structure. Electron microscopy revealed an abundance of microfilaments and primary lysosomes in the cytoplasm, and myelin figures were observed in tumor cells (Fig. 1b). The results indicated that tumor cell numbers were increased and not dead after chemotherapy and that tumor cell autophagy was activated.

### Autophagy is increased and apoptosis is reduced in the EOC re-ascites group, resulting in chemoresistance

**Fig. 2:**
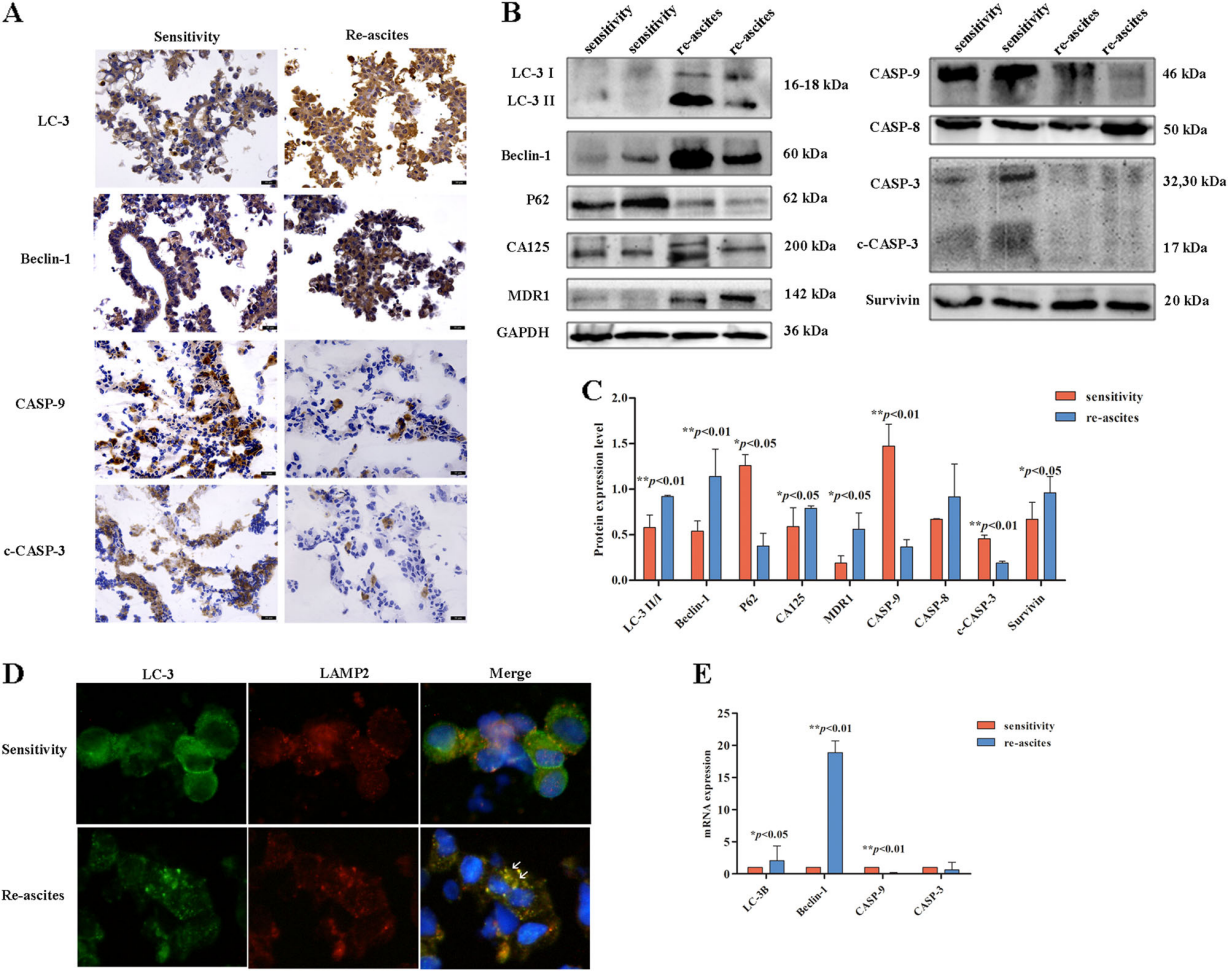
Autophagy is increased and apoptosis is reduced in the EOC re-ascites group. **a** IHC analysis of LC-3, Belin-1, CASP-9, and c-CASP-3 expression in EOC ascites cells (scale bar = 30 µm). **b**, **c** Western blot analysis of autophagy/apoptosis-related proteins expressed in the chemosensitive group and re-ascites group (***p < 0.001, **p < 0.01, *p < 0.05). **d** Immunofluorescence analysis of the chemosensitive group and re-ascites group with LC3 (green) and LAMP2 (red) staining. Nuclei were counterstained with DAPI (blue). **e** qRT-PCR analysis of the average relative mRNA expression levels of autophagy/apoptosis-related genes in the chemosensitive group and re-ascites group (**p < 0.01, *p < 0.05)

Whether tumor cell growth in the re-ascites group was correlated with the change in autophagy/apoptosis level was evaluated by comparison with the sensitive group. IHC showed that LC-3 and Beclin-1 expression levels were increased in the re-ascites group; in contrast, CASP-9 and c-CASP-3 expression levels were reduced (Fig. 2a). Compared with the sensitive group, Western blot analysis showed that CA125, MDR1, LC-3, Beclin-1, and survivin expression levels were significantly increased, and P62, CASP-9, and c-CASP-3 expression levels were significantly decreased, while CASP-8 was non-significantly changed in the re-ascites group (Fig. 2b, *p* < 0.01, *p* < 0.05). IF showed that co-expression of LC3 and LAMP2 was increased in the re-ascites group compared to the chemosensitive group (Fig. 2c). Then, mRNA levels were detected by qRT-PCR, and the results showed that LC-3B and Beclin-1 expression levels were increased, while the CASP-9 expression level was decreased in the re-ascites group compared to the chemosensitive group (Fig. 2d, *p* < 0.01, *p* < 0.05). The analysis showed that compared with the sensitive group, autophagy activity and the level of MDR1 were increased and apoptosis was decreased in the re-ascites group.

### Identification of tumor cell components in precipitated EOC ascites cells

**Fig. 3:**
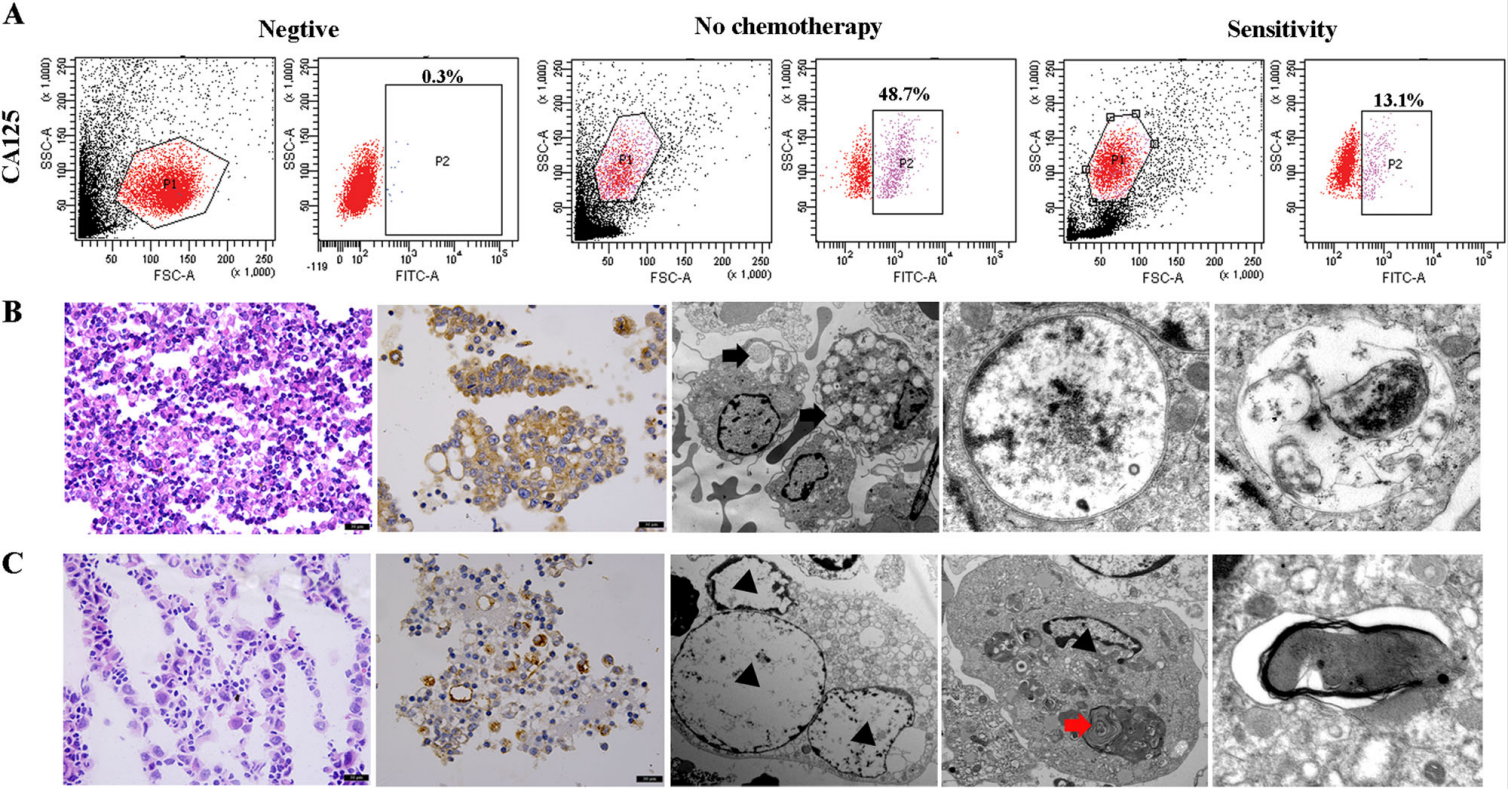
Evaluation of the precipitated EOC ascites cells. **a** The tumor cell content in EOC ascites cells, and CA125/FITC staining of precipitated EOC ascites tumor cells. **b** Light microscope and electron microscope images from the no-chemotherapy group. Numerous autophagic vacuoles were noted in tumor cells, and the composition of organelles and floc in autophagic vacuoles was observed (arrow in black). **c** Light microscope and electron microscope images from the chemosensitive group. In tumor cells, apoptotic nuclei (triangle) were observed, the number of autophagosomes was decreased, karyopyknosis was detected, and autophagosomes exhibited medullary alterations (arrow in red)

To confirm that ascites cells can reflect changes in tumor cells, we identified tumor cell components in precipitated EOC ascites cells. Flow cytometry results indicated that the tumor cell content in precipitated EOC ascites cells was 48.7% in the no-chemotherapy group and 13.1% in the sensitive group (Fig. 3a). In the no-chemotherapy group, tumor cells were large and dense, and CA125 was highly expressed. In addition, electron microscopy showed that more autophagosomes or autophagic vacuoles were present in tumors cells of the no-chemotherapy group (Fig. 3b). In contrast, in the sensitive group, tumor cell numbers were decreased, and the expression level of CA125 was also reduced compared to the no-chemotherapy group. Electron microscopy revealed that the number of autophagosomes was decreased in the sensitive group compared to the no-chemotherapy group and that some autophagosomes exhibited medullary alteration (Fig. 3c).

### Autophagy is reduced in the EOC sensitive group

**Fig. 4:**
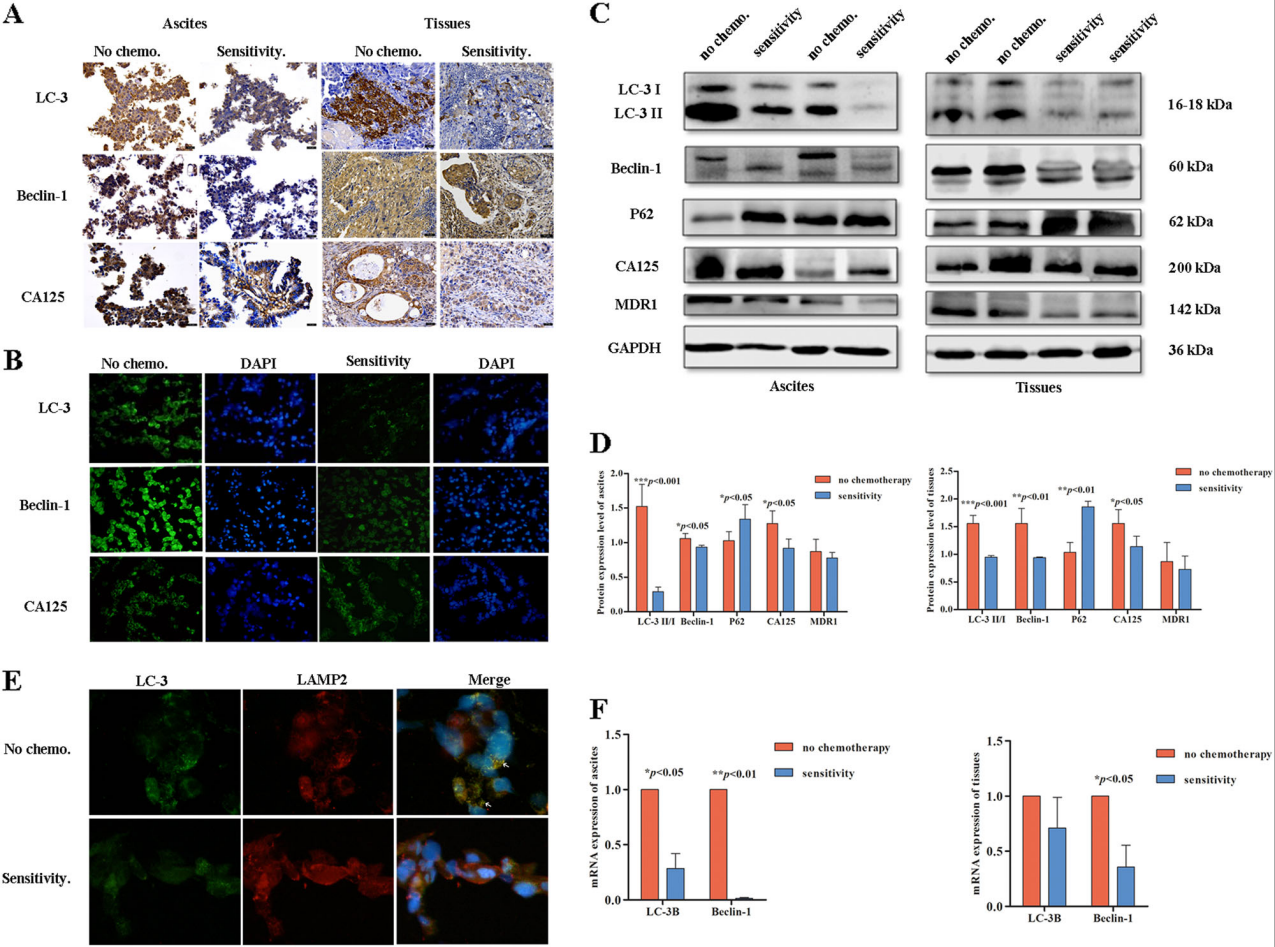
Autophagy is reduced in the EOC chemosensitive group. **a**, **b** IHC and IF analysis of autophagic protein expression in ovarian cancer samples. **c,d** Western blot analysis of autophagy proteins expressed in the no-chemotherapy group and chemosensitive group (***p < 0.001, **p < 0.01, *p < 0.05). **e** Immunofluorescence analysis of the no-chemotherapy group and chemosensitive group with LC3 (green) and LAMP2 (red) staining. Nuclei were counterstained with DAPI (blue). **f** qRT-PCR analysis of the average relative mRNA expression levels of autophagy-related genes in the no-chemotherapy group and chemosensitive group (**p < 0.01, *p < 0.05).

After chemotherapy, the number of tumor cells was significantly decreased in ovarian cancer samples (ascites cells and tissues). IHC was performed on ascites cells and tissues, and the results indicated that the sensitive group expressed lower levels of LC-3, Beclin-1, and CA125 compared with the no-chemotherapy group (Fig. 4a). IF analysis of ascites cells revealed similar results as the IHC analysis (Fig. 4b). Western blot analyzes of ascites cells and tissues indicated that LC-3, Beclin-1, and CA125 expression levels were significantly reduced after chemotherapy and that the expression level of P62 was increased after chemotherapy (Fig. 4c–d, *p* < 0.001, *p* < 0.01, *p* < 0.05). Co-immunofluorescence detection showed that LC3 and LAMP2 were co-expressed in the no-chemotherapy group but not in the sensitive group (Fig. 4e). The qRT-PCR results revealed that in the sensitive group, LC-3B and Beclin-1 mRNA levels were reduced in ascites cells (*p* < 0.05), and Beclin-1 levels were also significantly decreased in tissues (Fig. 4f, *p* < 0.05). This finding suggests that autophagy was significantly decreased in the EOC ascites cells and tissues in the sensitive group.

### Apoptosis is increased in the EOC sensitive group

**Fig. 5:**
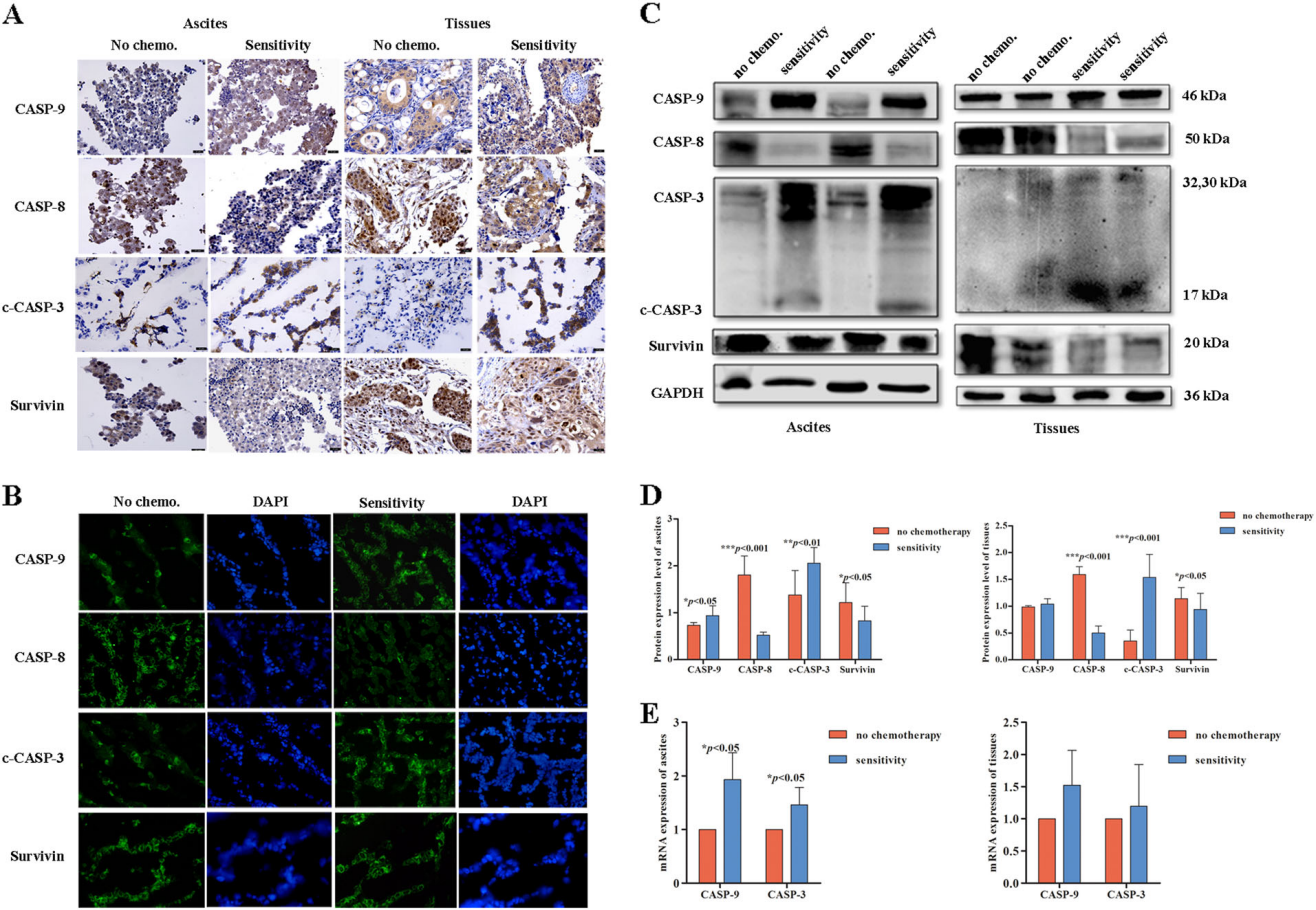
Apoptosis is increased in the EOC chemosensitive group. **a,b** IHC and IF analysis of apoptotic protein expression in ovarian cancer samples. **c,d** Western blot analysis of apoptotic proteins expressed in the no-chemotherapy group and chemosensitive group (***p < 0.001, **p < 0.01, *p < 0.05). **e** qRT-PCR analysis of the average relative mRNA expression levels of apoptosis-related genes in the no-chemotherapy group and chemosensitive group (*p < 0.05)

The level of apoptosis was detected in ovarian cancer samples (ascites cells and tissues). Ascites cells and tissues were evaluated by IHC, and the results indicated that the sensitive group expressed higher levels of CASP-9 and c-CASP-3 than the no-chemotherapy group, whereas CASP-8 and survivin expression levels were slightly decreased in the sensitive group compared with the no-chemotherapy group (Fig. 5a). IF analysis of ascites cells revealed similar results as the IHC analysis (Fig. 5b). In addition, Western blot analysis found that the mean expression levels of CASP-8 and survivin were reduced in the sensitive group compared with the no-chemotherapy group. In contrast, CASP-9 and c-CASP-3 expression levels were increased, and statistical analysis revealed a significant difference (Fig. 5c–d, *p* < 0.001, *p* < 0.01, *p* < 0.05). The qRT-PCR results of ascites revealed a significant increase in CASP-3 and CASP-9 mRNA levels in the sensitive group compared with the no-chemotherapy group (Fig. 5e, *p* < 0.05). This finding suggested that apoptosis was significantly increased in EOC ascites cells and tissues of the sensitive group compared to those of the no-chemotherapy group.

### Level of autophagy in EOC cell lines affects its sensitivity to chemotherapy drugs

**Fig. 6:**
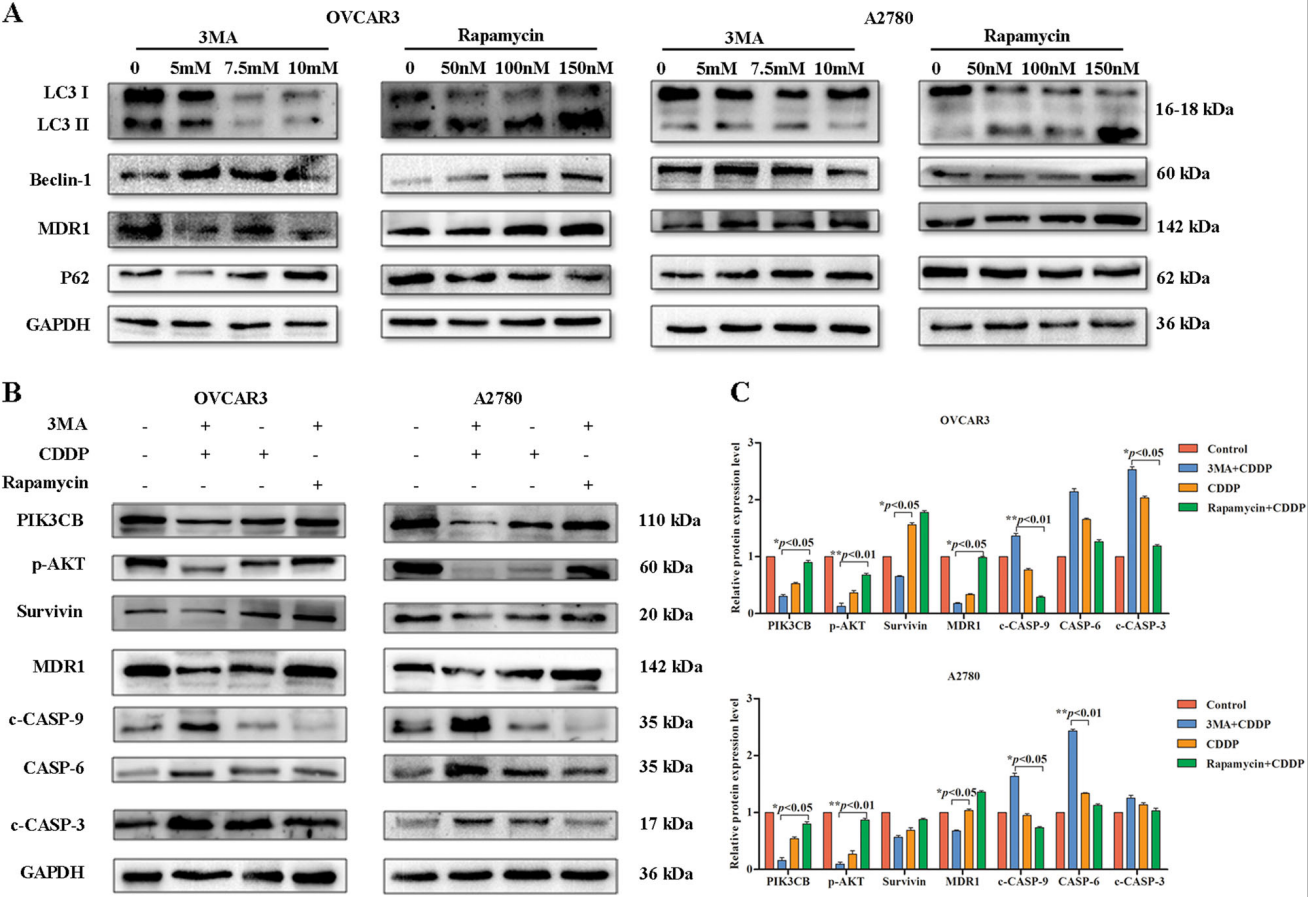
Level of autophagy in OVCAR3 and A2780 cells affects their sensitivity to chemotherapy drugs. **a** Western blot analysis of autophagy protein and MDR1 expression in the 3MA group and rapamycin group. **b,c** Western blot analysis of proliferation- and apoptosis-related proteins expressed in the 3MA+CDDP group, CDDP group, and rapamycin+CDDP group (**p < 0.01, *p < 0.05)

Autophagy was significantly reduced and apoptosis was significantly increased in the sensitive group compared with the no-chemotherapy group, suggesting that autophagy/apoptosis may accurately reflect the efficacy of chemotherapy. However, autophagy was increased and apoptosis was decreased in the re-ascites group, suggesting that the cause of re-ascites may be related to increased autophagy and decreased apoptosis, as increased autophagy inhibits cell death and promotes drug resistance. We performed a preliminary validation study using cell lines. The ovarian cancer cell lines OVCAR3 and A2780 were treated with 3MA (5, 7.5, and 10 mM) and rapamycin (50, 100, and 150 nM). Western blotting results showed that in the 3MA group, as the drug concentration increased, the protein levels of LC3II, Beclin-1 and MDR1 decreased, and P62 levels increased. The opposite results were observed in the rapamycin group (Fig. 6a). Subsequently, OVCAR3 and A2780 cells were pretreated with or without 3MA (10 mM) and rapamycin (150 nM), and then, they were treated with cisplatin (CDDP) (20 μM) for 24 h. Compared with CDDP group, cells that were pretreated with rapamycin followed by CDDP revealed significantly increased levels of p-AKT, PIK3CB, and MDR1 and decreased levels of c-CASP 9, c-CASP 3, and CASP 6. However, the 3MA+CDDP group exhibited the opposite results (Fig. 6b–c, *p* < 0.01, *p* < 0.05). This finding indicates that the level of autophagy may affect the sensitivity of A2780 and OVCAR3 cells to chemotherapy drugs. Inhibition of autophagy can increase the sensitivity of ovarian cancer cells to drugs and promote cell death. Meanwhile, inducing autophagy can reduce the sensitivity of ovarian cancer cells to chemotherapy drugs, inhibit cell death and promote drug resistance.

## Discussion

Autophagy plays an important role in regulating the homeostasis of cellular protein metabolism. Autophagy may promote tumor suppression by preventing tumor formation and promoting cell death, thereby limiting the number of tumor cells or reducing the probability of DNA mutation. On the other hand, autophagy may promote tumor protection by protecting cells from chemotherapeutic drugs and by slowing tumor cell apoptosis^2,16^. EOC patients commonly undergo removal of ascites by surgery and chemotherapy, which reduce or inhibit re-ascites; nevertheless, some patients still experience re-ascites. In the present study, we found that the level of autophagy in the precipitated ascites tumor cells was significantly higher in the re-ascites group than in the chemosenstive group and that autophagy may increase the survival ability of the cells. ELISA was used as a supplemental method to confirm that the results from the ascites supernatants were consistent with those from the ascites precipitates (Fig. S2). Moreover, studies have indicated that autophagy may cause drug resistance^17^. Meanwhile, we preliminarily confirmed in cell lines that inhibition of autophagy enhances CDDP-induced apoptotic cell death, whereas induction of autophagy protects tumor cells from CDDP-induced cell death, thus inducing cisplatin resistance. Zhang et al. reported that 3-MA converts the antagonistic effect from combined treatment of FTY720 with CDDP into an additive effect toward inducing ovarian cancer cell death^18^. Bao et al. confirmed that Nrf2 induced cisplatin resistance through the activation of autophagy in ovarian carcinoma^19^. These findings are consistent with our results. Studies using ovarian cancer cell lines have also found that the levels of autophagy were significantly higher in A2780cp cells (cisplatin-resistant ovarian carcinoma cells) than in A2780 cells (parental cell line) and that inhibition of autophagy by siRNA knockdown of Beclin-1 expression enhanced cisplatin-induced cell death and apoptosis^20^. Meanwhile, inhibition of the expression level of autophagy-related gene Beclin1 in SKOV3/DDP cells may increase the rate of apoptosis and increase their sensitivity to chemotherapeutic drugs^21^. Moreover, a series of studies by Shepherd and colleagues showed that the combination of AKT inhibition and autophagy blockade may effectively reduce the number of residual EOC cells that can contribute to ovarian cancer recurrence^22–24^. Levy JMM and others suggest that autophagy has opposing, context-dependent roles in cancer, and interventions to both stimulate and inhibit autophagy have been proposed as cancer therapies^25^. However, once cancer has developed, active autophagy can promote the growth of tumor cells, while autophagy inhibitors may elicit an anti-tumor effect^25^. Autophagy plays a protective role in tumor cells of re-ascites, and targeting autophagy by specifically regulating the autophagy pathway may be a top priority of EOC ascites researchs in the future. We have further studied the mechanism by which autophagy increases drug resistance by targeting miRNA in ovarian cancer (unpublished). In summary, autophagy has an inhibitory effect on tumorigenesis in early-stage malignant tumors. However, during tumor progression, the advantages and disadvantages of modulating autophagy to intervene with tumor cell growth should be further clarified and paid more attention.

Multidrug resistance (MDR) in tumor cells is an almost universal phenomenon in clinical treatment of tumors. MDR not only can directly lead to the failure of tumor treatment but also is closely related to tumor recurrence. Cisplatin is a common clinical chemotherapy drug for ovarian cancer and ovarian cancer with ascites. Cisplatin kills tumor cells by inhibiting the replication of DNA to induce cell apoptosis^26^. This study also confirmed that the ovarian cancer samples (ascites cells and tissues) from the sensitive group expressed higher levels of apoptosis-related genes than the no-chemotherapy group, which indicated that apoptosis could reflect tumor cell death and the efficacy of chemotherapy. Cisplatin resistance can result from epigenetic changes at molecular and cellular levels, including reduced accumulation of the platinum compounds, increased levels of DNA damage repair, alterations of membrane protein trafficking, and inactivation of the apoptosis pathway^27^. Inactivation of apoptosis is an important cause of chemotherapy resistance. Comparison of the sensitive group with the re-ascites group via IHC, Western blot, qRT-PCR, ELISA, and other methods showed that in the re-ascites group, the level of apoptosis was reduced and the level of MDR1 was increased, which may lead to drug resistance. Clinicopathological data revealed that serum CA125 levels were increased and that MDR was positively expressed in the re-ascites group. qRT-PCR also confirmed that MDR1 levels were higher in the re-ascites group than in the sensitive group. These results suggest that a large number of tumor cells develop resistance to chemotherapy drugs. In addition, it has been reported that MDR1/Pgp may be used to predict the clinical outcome of patients with advanced ovarian cancer^28^. The expression level of MDR1 in patients who did not respond to chemotherapy was significantly higher than that in patients who responded to chemotherapy^28^, which is consistent with our results. Various studies have shown that alterations in the apoptosis pathway that are indirectly induced by certain signaling pathways can prevent chemotherapy resistance^29–31^.

As a survival mechanism, autophagy maintains the integrity of cells by replenishing metabolic precursors and removing subcellular debris under stress conditions. By preventing the toxic accumulation of damaged protein and organelles, particularly mitochondria, autophagy limits oxidative stress, chronic tissue damage, and oncogenic signaling, thereby suppressing cancer initiation^9^. However, a key mechanism underlying how autophagy promotes the growth and survival of various cancers is its ability to support cellular metabolism^12^. The diverse metabolic fuel sources that can be produced by autophagy allow metabolic plasticity in tumors^9,32^. In summary, inhibition of autophagy may be a potential therapeutic modality to reverse anti-tumor drug resistance.

The study of ovarian cancer ascites and cell lines further confirms that increased autophagy can inhibit cell death and promote drug resistance in EOC cells. The results showed that in the no-chemotherapy group, a high expression level of Beclin-1, an autophagy marker, and  LC-3I turned to LC-3II, thereby increasing LC-3II expression levels and subsequently increasing the level of autophagy and decreasing the level of apoptosis. Then, in the sensitive group, the expression level of survivin, an inhibitor of apoptosis, is decreased, while the expression level of CASP-9, an effector of apoptosis, is increased, subsequently triggering the caspase cascade and leading to c-CASP-3 activation and enhanced tumor cell apoptosis. The level of MDR1, Beclin-1, and LC-3II expression was increased in the re-ascites group, which also suggested that autophagy was increased while apoptosis was decreased. Therefore, tumor cells are protected from death and mass proliferation, which lead to re-ascites in EOC patients (Fig. S3). Taken together, in the re-ascites group, activation of autophagy protects the tumor cell from the chemotherapy agent, and inhibition of apoptosis increases chemotherapy resistance in EOC patients. Therefore, reducing autophagy and promoting apoptosis, which increase the sensitivity of cancer cells to chemotherapeutics, may be a new treatment modality for ascites. Our study findings also provide a new strategy for the targeted therapy of ovarian cancer and support the potential development of specific autophagy regulators.

## Materials and methods

### Collection and processing of patient samples

One hundred and twenty-one cases of malignant ascites that were obtained from January 2014 to May 2017 from the Harbin Medical University Affiliated Cancer Hospital were included in this study. Among them, there were 105 cases of ovarian cancer and 16 cases of other tumors. Of the 105 cases of ovarian cancer ascites, 60 cases were treated without chemotherapy, and 45 cases were treated with chemotherapy. We also acquired 35 samples of fresh ovarian cancerous tissue, including 26 cases of serous ovarian cancer and 9 cases of mucinous ovarian cancer. All cases had complete clinical and pathological data (Table S1). According to the screening criteria for *Ovarian cancer, version 3.2012*. published by NCCN^33^. The patients who underwent chemotherapy were further divided into the chemosensitive (sensitive) group (25 cases) and the chemoresistant (re-ascites) group (20 cases). A flow diagram describing the study and groups are shown in Fig. S1 and Table S2.

The supernatants of cancerous ascites were stored at −80 °C for subsequent analysis by ELISA. Then, moderate ACK lysis (Leagene Biotechnology, China) buffer was added to the precipitates, which were collected into 1.5-mL centrifuge tubes. Subsequently, RNA, electron microscopy, light microscopy, and protein analyzes were performed.

### Ovarian carcinoma cell lines and culture

Two ovarian cancer cell lines (OVCAR3 and A2780) were obtained from the Cell Bank at the China Academy of Sciences (Shanghai, China). Cells were maintained in RPMI-1640 complete medium supplemented with 2 mM glutamine and 10% fetal bovine serum (FBS) at 37 °C in a humidified atmosphere containing 5% CO_2_. Using the previously described cytotoxicity assay, we found that the IC50 of A2780 and OVCAR3 to cisplatin was, respectively, 30 μM and 45 μM.

### Histopathological analysis techniques

Ovarian cancer tissues or precipitated ascites cells were routinely processed into paraffin blocks and then sectioned at a thickness of 4 to 6 µm. The sections were then stained with H&E. Precipitated ascites cells were fixed for 2 h in 2.5% glutaraldehyde in PBS (pH 7.4) and then fixed in 1% osmium tetroxide (pH 7.4) for 2 h. Samples were dehydrated in a graded series of acetone and then embedded in Epon 812. Samples were cut into ultrathin sections (50–70 nm in thickness), double-stained with uranyl acetate and lead citrate and examined with an electron microscope (H-7650).

### Flow cytometry

The levels of CA125 in the ascites of patients who were and were not treated with chemotherapy were detected. After cell counts were obtained, 1 × 10^6^ cells were added to a 1.5-mL tube. Cells were then rinsed once with staining buffer, and 1 µL of CA125 (Bioss, bs-0091R) antibody was added (staining buffer was used as a negative control) for a total volume of 100 µL. After fixing the cells for 30 min in the dark in 500 µL of 1% paraformaldehyde on ice, the detection assay was performed. This assay was repeated thrice.

### Immunofluorescence (IF) and immunohistochemistry (IHC)

Frozen sections of cell components were routinely generated to detect Beclin-1 (Abcam, ab114071, 1:400), LC-3 (Elabscience, EPP14859, 1:100), LAMP2 (Proteintech, 66301-1-lg, 1:50), CA125 (Bioss, bs-0091R, 1:25), c-CASP-3 (Abcam, ab32042, 1:100), CASP-9 (Affinity, AF6348, 1:100), CASP-8 (Elabscience, EPM12180, 1:200), and survivin (Affinity, AF6017, 1:50) by IF. Images were then obtained using a Nikon E800 Multifunctional Biological Microscope and Image Acquisition Software Nikon ACT 21 version 6.1.

Beclin-1 (1:300), LC-3 (1:100), CA125 (1:50), c-CASP-3 (1:200), CASP-9 (1:100), CASP-8 (1:300), and survivin (1:50) were detected in paraffin sections of cell precipitates. Thirty-five ovarian cancer tissue samples were paraffin-embedded, and Beclin-1 (1:200), LC-3 (1:100), CA125 (1:100), c-CASP-3 (1:100), CASP-9 (1:50), CASP-8 (1:150), and survivin (1:50) were detected in the ovarian cancer tissues using the sABC method. Descriptions of the specific steps are described elsewhere.

### Western blot

Total protein from cell lines, precipitated ascites cells and ovarian cancer tissues was extracted, and the protein concentration was determined using a BCA protein concentration detection kit. Equal amounts of cellular proteins were loaded into each well and resolved using 15% SDS-PAGE gels. Polyvinylidene fluoride membrane blotting was subsequently performed under standard conditions. The following primary antibodies were used in this experiment: Beclin-1, LC-3, P62 (Proteintech, 18420-1-AP, 1:1000), CA125, MDR1 (Affinity, AF5185, 1:500), survivin, c-CASP-3, CASP-9, CASP-8, p-AKT (Affinity, AF0016, 1:1000), PIK3CB (Omnimabs, OM288097, 1:1000), and GAPDH. The GAPDH (Tianjin Sungene Biotech Ltd., KM9002) antibody was diluted 1:5000, and the CA125 antibody was diluted 1:500. The other primary antibodies were diluted 1:1000 and incubated with the membrane overnight at 4 °C. Subsequently, a 1:5000 dilution of the secondary antibody was added to the membrane. Then, the blot was developed with an ECL kit.

### Quantitative real-time-PCR (qRT-PCR)

Total RNA was isolated and converted to cDNA using a cDNA synthesis kit (Transgen Biotech, AT311). Amplification of cDNA was performed in a total volume of 20 µL of Tip Green qPCR (Transgen Biotech, QA141) using a Roche LightCycler 96. Primer sequences for quantitative PCR, which were synthesized by GENEWIZ, are listed in Supplementary Table S3.

### Statistical analysis

Statistical analyzes were performed using SPSS 17.0 software. Spearman’s test was used to evaluate the associations between autophagy and apoptosis. One-way analysis of variance (ANOVA) was used to analyze the differences between groups. An independent *t*-test or *u*-test for differences in the mean values was used for comparison. Comparisons between two or multiple parameters were performed using the chi-square test. A *p* < 0.05 was considered statistically significant, and values of *p* < 0.01 or *p* < 0.001 were considered highly statistically significant. GraphPad Prism was used for mapping and curve fitting.

## Electronic supplementary material

Figure S1(TIF 944 kb)

Figure S2(TIF 1714 kb)

Figure S3(TIF 1345 kb)

Supplementary Table S1-S3(DOC 47 kb)

Supplementary Information(DOC 25 kb)
