# Supplementary figures and images for: Increased autophagy in EOC re-ascites cells can inhibit cell death and promote drug resistance
Source: Cell Death Dis. 2018 Mar 16;9(4):419. doi: 10.1038/s41419-018-0449-5 (PMC5856849; doi:10.1038/s41419-018-0449-5)

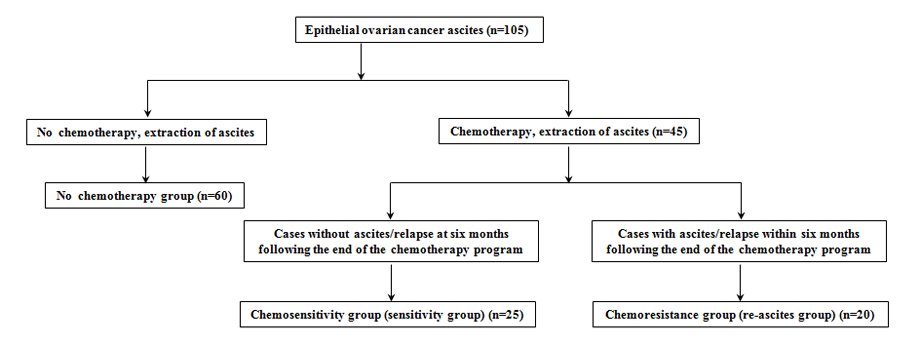

Supplement: Supplementary file 1 — Figure S1(TIF 944 kb) [file 41419_2018_449_MOESM1_ESM.tif]

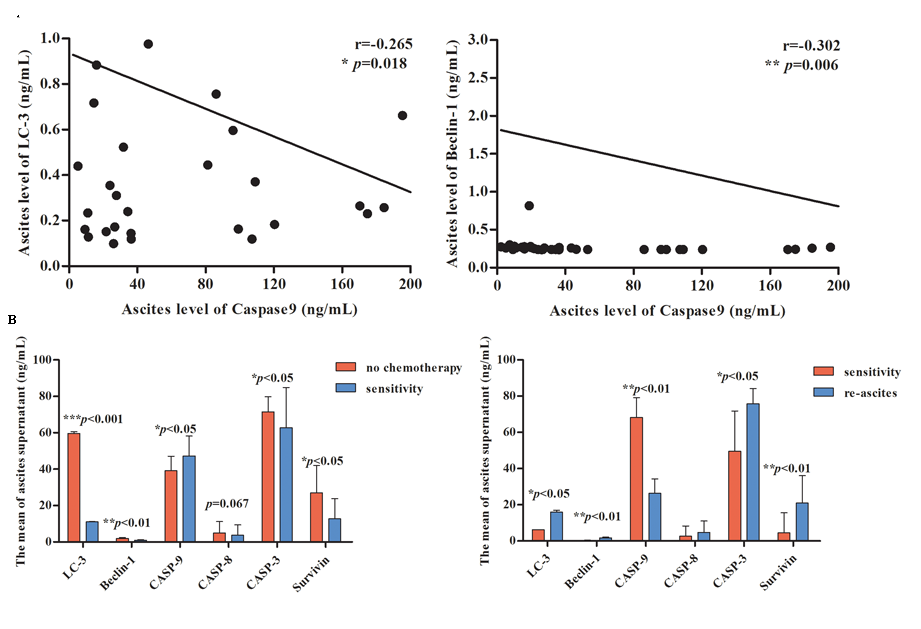

Supplement: Supplementary file 2 — Figure S2(TIF 1714 kb) [file 41419_2018_449_MOESM2_ESM.tif]

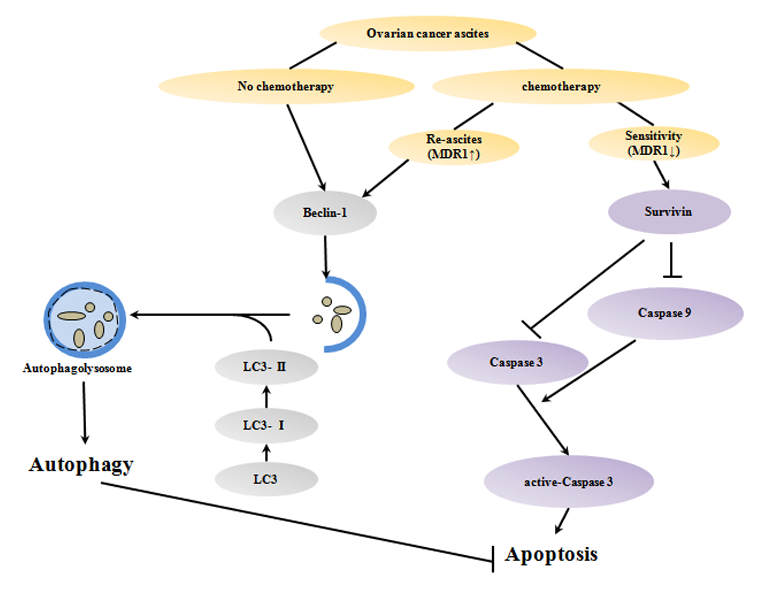

Supplement: Supplementary file 3 — Figure S3(TIF 1345 kb) [file 41419_2018_449_MOESM3_ESM.tif]
