# Supplementary material for: Increased autophagy in EOC re-ascites cells can inhibit cell death and promote drug resistance
Source: Cell Death Dis. 2018 Mar 16;9(4):419. doi: 10.1038/s41419-018-0449-5 (PMC5856849; doi:10.1038/s41419-018-0449-5)
Supplement: Supplementary file 4 — Supplementary Table S1-S3(DOC 47 kb) [file 41419_2018_449_MOESM4_ESM.doc]

Table S1.The clinicopathological data of EOC patients

|  | n=105(%) |  |
| --- | --- | --- |
| Age |  |  |
| ≤50 | 34(32.4) |  |
| >50 | 71(67.6) |  |
| Chemotherapy |  |  |
| No chemotherapy | 60(57.1) |  |
| Chemotherapy | 45(42.9) |  |
| Histological type |  |  |
| Serous | 88(83.8) |  |
| Mucinous and others | 17(16.2) |  |
| Differentiation |  |  |
| Well | 13(12.4) |  |
| Moderate | 26(24.7) |  |
| Poor | 66(62.9) |  |
| Lymphatic metastasis |  |  |
| No | 75(71.4) |  |
| Yes | 30(28.6) |  |

Table S2. Samples of EOC ascites and tissues

|  | EOC ascites (n=105) | EOC tissues (n=35) |
| --- | --- | --- |
| No chemotherapy group | 60 | 14 |
| Chemosensitivity group | 25 | 13 |
| Chemoresistance (re-ascites) group | 20 | 8 |

Table S3. Sequences of primers used for quantitative real-time-PCR

| Gene name | Primer Sequences |
| --- | --- |
| Beclin-1 | Forward: GACAGTGAACAGTTACAGATGG |
|  | Reverse: TCAGCCTGGACCTTCTCG |
| LC-3B | Forward: GAGAAGCAGCTTCCTGTTCTGG |
|  | Reverse: GTGTCCGTTCACCAACAGGAAG |
| CASP-3 | Forward: AGAACTGGACTGTGGCATTG |
|  | Reverse: CACAAAGCGACTGGATGAAC |
| CASP-9 | Forward: TTCCCAGGTTTTGTCTCCTG |
|  | Reverse: GGGACTGCAGGTCTTCAGAG |
| GAPDH | Forward: GGCCTCCAAGGAGTAAGACC |
|  | Reverse: AGGGGTCTACATGGCAACTG |
