# Supplementary material for: Increased autophagy in EOC re-ascites cells can inhibit cell death and promote drug resistance
Source: Cell Death Dis. 2018 Mar 16;9(4):419. doi: 10.1038/s41419-018-0449-5 (PMC5856849; doi:10.1038/s41419-018-0449-5)
Supplement: Supplementary file 5 — Supplementary Information(DOC 25 kb) [file 41419_2018_449_MOESM5_ESM.doc]

**Supplementary materials**

**Figure S1. A flow diagram describing the study and groups.**

**Figure S2. The results from the ascites supernatants.**

**Figure S3. Interacted relation of autophagy/ apoptosis-related proteins in ovarian cancer ascites.**
